# Evaluation of a New Culture-Based AtbFinder Test-System Employing a Novel Nutrient Medium for the Selection of Optimal Antibiotics for Critically Ill Patients with Polymicrobial Infections within 4 h

**DOI:** 10.3390/microorganisms9050990

**Published:** 2021-05-04

**Authors:** George Tetz, Victor Tetz

**Affiliations:** Human Microbiology Institute, New York, NY 10013, USA; info@hmi-us.com

**Keywords:** antibiotic resistance, antimicrobial susceptibility testing, novel diagnostics, polymicrobial, sputum, drug resistant

## Abstract

Here, we describe the validation of a new phenotypic culture-based AtbFinder method for rapid selection of antibiotics in vitro using specimens with mono- and polybacterial infections. AtbFinder, which can be applied to any type of non-blood tissue, does not require isolation of pure bacterial cultures. The method uses a novel TGV medium that allows more rapid bacterial growth of Gram-positive and Gram-negative monoisolates compared with that achieved with conventional laboratory media, demonstrating overall sensitivity, specificity, PPV, NPV values of 99.6%, 98.1%, 98.5%, and 99.4%, respectively, after 4 h. For polymicrobial infections, AtbFinder utilized a novel paradigm of the population response to antibiotics, enabling bacterial growth in the form of a mixed microbial community and selecting antibiotics targeting not only the principal pathogen, but also those bacteria that support their growth. TGV medium allowed culturing of a more diverse set of bacteria from polymicrobial biospecimens, compared with that achieved with the standard media, and enabled, within 4 h, accurate selection of the antibiotics that completely eliminated all cultivatable bacteria from clinical samples. In conclusion, the AtbFinder system may be a valuable tool in improving antibiotic selection, and enabling targeted empirical therapy and accurate antibiotic replacement, which is especially important in high-risk patients.

## 1. Introduction

Antibiotic therapy is typically started on an empirical basis, because the causative organism is not identified in an appreciable proportion of patients [1,2,3]. However, empirical antibiotic therapy is inadequate in over 25% of cases, with 8–12% of patients receiving antibiotics that are ineffective [4]. For these patients, antibiotic therapy must be adjusted following antimicrobial susceptibility testing (AST) by culture-based and/or molecular biology methods. However, even following the switch of the empirically chosen antimicrobial, in over 10% of cases, the newly selected antimicrobial remains ineffective [3]. The resulting clinical failure is particularly dangerous for immunocompromised patients.

There are several types of diagnostic tests used currently for the selection of appropriate antibiotics, but none of them is ideal with regard to expedience and accuracy [5]. Currently, traditional methods based on the isolation of bacterial culture require at least 24 h to detect the growth of bacteria isolated from clinical specimens after sampling and another 6 to 24 h for detailed isolate characterization (i.e., biochemical identification) or susceptibility testing, during which time the treatment can only be empirical [6,7]. Therefore, culture-based methods, owing to the slow turnaround time of results, are not suitable for tailored empirical therapy and are suboptimal in acute infection cases.

Another concern with culture-based tests is that media used in classical AST techniques, such as LB, Columbia and others, do not allow the growth of complex polymicrobial communities [8,9]. As a result, some clinically relevant organisms that are more difficult to culture do not get detected by standard AST of biological samples, leading to therapy failure [9]. Many contemporary methods allow isolation of only the predominant species, whereas bacteria at the site of infection are rarely represented by a single species [10]. The vast majority of the infections of the respiratory tract, urinary tract, skin, and soft-tissues had been previously characterized as being monomicrobial due to inaccurate results from culture-dependent isolation techniques. In contrast, the use of more advanced methods today indicates their polymicrobial nature [8,11,12,13,14]. Bacteria within polymicrobial communities are significantly more resistant to antimicrobials as they share collective antibiotic resistance [15]. Furthermore, they are characterized by combined virulence contributing to the increased mortality rate among critically ill and compromised patients, such as patients with HIV or those that underwent solid organ or marrow transplantation [10,16,17,18].

Another reason for the inappropriate selection of antibiotics by the standard AST methods is that they focus only on well-known pathogenic bacteria [8,19]. However, many bacteria that are considered nonpathogenic in non-immunocompromised patients may be pathogenic in people with suppressed immune response [20]. This may happen because, even in the case of successful elimination of one specific pathogen with a selected antibiotic, other bacteria that remain at the site of infection continue to grow and result in disease progression. Moreover, there is no benchmark accurate enough to indicate with certainty that bacteria are definitely pathogenic [21]. For example, *Pandoraea apista*, *Aeromonas* spp., and *Kluyvera* spp. are now classified as pathogenic, although several decades ago, they were believed to be opportunistic and safe microorganisms [21,22,23,24]. The media used in standard AST mostly promote the growth of the predominant bacteria and do not necessarily support proliferation of minor bacterial species from biological samples that may depend on each other for growth [25]. That is why, it has been estimated that less than 10% of bacteria can be cultured in laboratory conditions and assayed by conventional AST [26,27,28].

In order to overcome these obstacles, in the last few decades, several types of novel diagnostic tests for rapid identification of bacteria and AST have been developed [29,30]. Such tests are based on direct pathogen identification using nucleic acid amplification techniques [5,31]. Although these methods are frequently utilized to determine the causative agents of bloodstream infections, some of them are used for the infections of other parts of the body [32,33,34]. These methods do not depend on in vitro growth for bacterial detection and have become routine in many clinical microbiology laboratories. The introduction of PCR assays and sequencing technologies shortened the turnaround time to obtain the results and enabled characterization of polymicrobial infections [35,36]. However, these assays also have a number of limitations. In particular, although the identification of bacteria helps to determine the therapeutic strategy, it does not really inform on the extent of resistance to antibiotics [6]. Moreover, the methods based on *16S RNA* sequencing are not very accurate as many bacterial species share similar or identical *16S rRNA* sequences [37,38].

Some molecular biology methods are based on the detection of antimicrobial resistance genes [39,40]. However, the reliance on the presence of resistance genes may be misleading with regard to accurate antibiotic selection as not all resistant genotypes result in resistant phenotypes [6]. Furthermore, these methods can only detect known antibiotic resistance genes but may omit those, which have not yet been discovered [39,41]. The available resistance markers are not sufficiently comprehensive to provide clinically actionable results and may lead to the usage of either unnecessarily broad spectrum antibiotics or those without the sufficiently strong therapeutic effect.

Consequently, culture-based AST that predicts not only the resistance but also susceptibility to different drugs is still considered the gold standard for the identification of the most appropriate antibiotic for various infections [42].

Here, we evaluated a novel culture-based AtbFinder system that utilizes the recently developed TGV medium that supports simultaneous growth of a significantly more diverse set of bacteria from biological samples compared with that achieved with conventional laboratory media and uses a novel paradigm for rapid phenotypic-based antibiotic selection for monobacterial and polybacterial clinical isolates.

The AtbFinder approach is based on a novel paradigm for the selection of effective antibiotics that considers not only monobacterial infection but also polybacterial cooperative interactions at the infection site.

## 2. Materials and Methods

### 2.1. Study Samples and Laboratory Settings

Study specimens were obtained from the Human Microbiology Institute (New York, NY, USA), maintained at about 4 °C (wet ice), but not frozen, and sent to TGV-Dx within 8 h after sampling.

Bronchoalveolar lavage (BAL) or sputum samples obtained from patients with chronic obstructive pulmonary disease (COPD), community-acquired pneumonia (CAP), ventilator-associated pneumonia (VAP), hospital-acquired pneumonia (HAP), and cystic fibrosis (CF) were tested by using both state-of-the-art methods and AtbFinder system.

### 2.2. Antibiotics

AST was limited to antibiotics used for the treatment of respiratory infections, which were selected according to the American Thoracic Society/Infectious Disease Society of America guidelines [43,44,45]. Antibiotics were added to TGV medium at respective maximal concentrations that could be achieved at the site of infection according to literature data: amikacin, 9 µg/mL [46]; azithromycin 8 µg/mL [46,47]; aztreonam 22 µg/mL [48,49,50]; cefepime 24 µg/mL [46,49,50]; gentamicin 5 µg/mL [46,51]; levofloxacin 12 µg/mL [46,52]; linezolid 25 µg/mL [53,54]; meropenem 10 µg/mL [55]; piperacillin-tazobactam (25 for piperacillin and 3.5 for tazobactam) µg/mL [56]; vancomycin 12 µg/mL [46,57] (all from Sigma-Aldrich, St. Louis, MO, USA). Probes were incubated at 37 °C, for 4, 8, and 24 h.

### 2.3. Bacterial Strains and Growth Conditions

Bacterial clinical isolates used as monobacterial cultures for the validation of bacterial population diversity and growth speed the TGV agar included the following Gram-positive and Gram-negative bacteria: *S. aureus* (*n* = 22), *Streptococcus pneumoniae* (*n* = 4), *Streptococcus epidermidis* (*n* = 4), *Streptococcus pyogenes* (*n* = 2), *Enterococcus faecalis* (*n* = 5)*, Bacillus cereues* (*n* = 4), *Paenibacillus VT400* (*n* = 1), *Bacillus respiratorii* VT-16-64 (*n* = 1), *Escherichia coli* (*n* = 15), *Acinetobacter baumannii* (*n* = 2), *Stenotropomonas maltophilia* (*n* = 4), *Klebsiella pneumoniae* (*n* = 10), *Proteus vulgaris* (*n* = 4), *Proteus mirabilis* (*n* = 5), *Haemophilus influenzae* (*n* = 1), *Klebsiella oxytoca* (*n* = 1), *Rothia mucilaginosa* (*n* = 1), *Moraxella catharrhalis* (*n* = 1) *Pseudomonas aeruginosa* (*n* = 16), *Burkholderia cenocepacia* (*n* = 6), *Enterobacter cloacae complex* (*n* = 6), *Achromobacter xylosoxidans* (*n* = 2), *Serratia marcescens* (*n* = 3).

Bacteria were obtained from the Cystic Fibrosis Foundation Therapeutics Development Network Resource Center for Microbiology at the Seattle Children’s Hospital (Seattle, WA, USA) and from the Human Microbiology Institute (New York, NY, USA). Individual patterns of resistance to antibiotics are summarized in Supplementary Table S1.

All bacterial strains were sub-cultured from frozen stocks onto Columbia broth (Oxoid Ltd., London, UK) and incubated at 37 °C overnight. A standard bacterial inoculum of 5 × 10^5^ CFU/mL was used. All subsequent liquid subcultures were derived from colonies isolated from these plates and further grown at different solid media: LB agar (Oxoid Ltd., London, UK), Columbia agar (Oxoid Ltd., London, UK), Brain heart infusion agar (Oxoid Ltd., London, UK), *Burkholderia cepacia*-selective agar (Hardy Diagnostics, Santa Maria, CA, USA), chocolate agar (Oxoid Ltd., London, UK), Mueller-Hinton broth (Oxoid Ltd., London, UK), CHROMagar Staph aureus Medium (Becton, Dickinson and Company, Franklin Lakes, NJ, USA), TGV medium (Human Microbiology Institute, New York, NY, USA). All media were supplemented with 5% lysed sheep erythrocytes (Becton Dickinson, Heidelberg, Germany).

### 2.4. Biological Specimen Processing and Bacterial Isolation 

This study was approved by the Institutional Review Board and Ethics Committee of Human Microbiology Institute (Number: 018-4-AF). BAL or sputum biospecimens (1 mL) from patients with ventilator-associated, community-acquired pneumonia, cystic fibrosis, or chronic obstructive pulmonary disease were mixed with 1 mL of sterile H_2_O until homogeneity with a plastic swab. After homogenization, 25 µL of the suspension was directly plated onto TGV agar in each well of a 12-well plate and incubated at 37 °C, for 4, 18, and 24 h. For the control arm, 100 µL suspension were plated on 90 mm Petri dish with LB agar, *Burkholderia cepacia*-selective agar (Hardy Diagnostics), chocolate agar (Oxoid) or CHROMagar Staph aureus Medium (Becton, Dickinson and Company) and cultured according to laboratory recommendations at 37 °C for 24–72 h [8]. The results of the study were not reported to the physician, and no medical decisions were made on the basis of these data.

### 2.5. Identification of Bacteria in Biological Specimens

Bacterial identification to the species level from biological specimens was done by using subcultures on AtbFinder or LB medium (L). Isolates were examined for purity by light microscopy (Leica 2500DM, Leica Microsystems, Wetzlar, Germany). To exclude the presence of mixed bacterial cultures, the isolates were assessed from at least 10 fields of view [58]. After the subcultivation of every mixed bacterial culture up to monocultures, subsequent biochemical identification and matrix assisted laser desorption/ionization-time of flight (MALDI-TOF) mass spectrometry (Microflex LT, Bruker Daltonics, Bremen, Germany) analysis were performed according to the manufacturer’s instructions after 24 h of growth.

The complete *16S rRNA* gene sequencing of bacterial colonies was also used to identify the isolates. A PCR was performed with the general bacterial primers 27f (5′-AGAGTTTGATCCTGGCTCAG-3′) and 1492r (5′-GGTTACCTTGTTACGACTT-3′). The PCR mixture contained 0.2 μM of each primer and 40 ng of bacterial DNA in a total volume of 100 µL (HotStar Taq Master Mix Kit, Qiagen, Valencia, CA, USA) [59].

PCR was performed with a Mastercycler EP S gradient thermocycler (Eppendorf, Germany) using the following protocol: one cycle of 15 min at 95 °C, followed by 30 cycles, of (60 s at 95 °C, 45 s at 50 °C, and 90 s at 72 °C), and the final extension cycle of 10 min at 72 °C. The PCR products were verified by electrophoresis in 0.8% agarose gel, and the rest of the sample was purified and concentrated in 30 µL of demineralized water using a Qiaquick PCR purification kit (Qiagen) [60].

PCR amplicons were sequenced with a BigDye Terminator v1.1 cycle sequencing kit (Life Technology, Foster City, CA, USA). Identification to the species level was conducted by comparing the obtained sequences with those in BLAST database (http://blast.ncbi.nlm.nih.gov/Blast.cgi (accessed on 10 March 2019)). Identifications to the genus and species-level were done on the basis of ≥97% and 99% identity of the *16S rRNA* gene sequence to the reference, respectively [61].

### 2.6. The AtbFinder System and Interpretation of Results

The AtbFinder system is a multi-well (12, 24, 48 or 96) (Sarstedt, Newton, NC, USA) filled with TGV medium that comprises pancreatic digests of casein, peptic digest of meat, heart pancreatic digest, yeast extract, starch, and water (Human Microbiology Institute, New York, NY, USA).

Clinical specimens were directly plated onto TGV agar in each well with a sterile swab, avoiding scratching or damaging the agar. Ten of the twelve wells contained TGV agar with antibiotics (one antibiotic per well) at concentrations that can be practically achieved at the site of infection. Two control wells contained antibiotic-free TGV agar. Plate reading was performed following sampling and incubation at 37 °C, for 4, 18, and 24 h.

The presence of microbial growth was identified with the naked eye and confirmed with a stereoscopic microscope (Leica S6, Leica Microsystems, Germany). The signs of early bacterial growth, namely hemolysis, appearance of film, and microcolonies, in the wells with antibiotics were compared with signs of bacterial growth in control wells.

Microbial growth in any well meant that in the biospecimens, there were microorganisms resistant to the particular antibiotic in the nutrient medium in the well; therefore, the antibiotic was categorized as “ineffective”. An absence of bacterial growth in the well meant that the antibiotic present in the well had killed or inhibited the growth of all bacteria in the biospecimens and was thus categorized as “effective”. In rare cases, there was no growth in control wells after 4 h, so those cultures were not analyzed further as is separately described in the Results below.

### 2.7. Gold Standard Definition

Culture-based in vitro AST was selected as the standard of care method. The minimal inhibitory concentrations (MICs) for antimicrobials were determined by the broth microdilution method according to the Clinical and Laboratory Standards Institute (CLSI) guidelines [62,63]. The isolates were categorized as susceptible or resistant according to CLSI breakpoint guidelines with the only modification that “intermediate” isolates were treated as “resistant” [64]. A standard bacterial inoculum of 5 × 10^5^ CFU/mL was used. Serial twofold dilutions of the antimicrobials were prepared in cation-adjusted Mueller-Hinton broth. MIC was defined as the lowest concentration of antibiotic that completely inhibited visible growth. Experiments were conducted in triplicate.

### 2.8. Data Analysis

The results of antibiotic selection from the AtbFinder system were compared to those obtained with the culture-based gold standard microdilution method. For the purpose of data analysis, the following definitions were used. A true-positive result occurred when both the AtbFinder and standard-of-care methods indicated that the microorganism was resistant to the antibiotic. A true-negative result occurred when both AtbFinder and standard-of-care methods demonstrated sensitivity of the microorganism to the antibiotic. A false-positive result occurred when the AtbFinder system identified a strain as being sensitive to a certain antibiotic, but according to the standard-of-care method, the organism was resistant to that antibiotic. A false-negative result was recorded when the AtbFinder system suggested that the microorganism was resistant to the antibiotic, whereas the standard-of-care method indicated that it was sensitive. We calculated the values of accuracy, sensitivity, specificity, as well as positive predictive value (PPV) and negative predictive value (NPV), as previously described [65].

The total category agreement (CA) was determined and the results which differed from those obtained by the state of care microdilution method were categorized as described previously [66]. Thus, the very major errors were recorded in the cases when the AtbFinder system indicated that an isolate was susceptible to the antibiotic, whereas according to the standard of care method, the isolate was resistant. The major errors were recorded in the cases when the AtbFinder system suggested that an isolate was resistant to the antibiotic, whereas the standard of care method indicated that the isolate was susceptible. The rate of very major errors was calculated by dividing the total number of very major errors by the total number of strains determined as being resistant and multiplied by 100%. The rate of major errors was determined by dividing the total number of major errors by the number of strains determined as being susceptible and multiplied by 100%.

### 2.9. Bacterial Diversity Analysis

The variety of bacteria that grew on the media used was characterized by the α-diversity indices such as non-parametric abundance-based coverage estimator (ACE) and Chao 1, indices that were managed and analyzed using R version 3.4.1 software [67].

Species richness (a count of different species) that resulted in growth on different media were represented on a dot plot, generated by package ‘ggplot2’ within R version 3.4.1 [67]. All statistical analyses were conducted with a significance level of α = 0.05 (*p* < 0.05).

### 2.10. Statistics

Two-way ANOVA comparisons test was applied within the same data sets to test difference between microbial growth on different media at each time point. GraphPad Prism version 9 (GraphPad Software, San Diego, CA, USA) or Excel 10 were applied for statistical analysis and illustration was used if not stated differently. *p*-values < 0.05 were significant.

## 3. Results

### 3.1. Comparison of Bacterial Growth Rate on TGV Medium to That on Other Media

**Figure 1:**
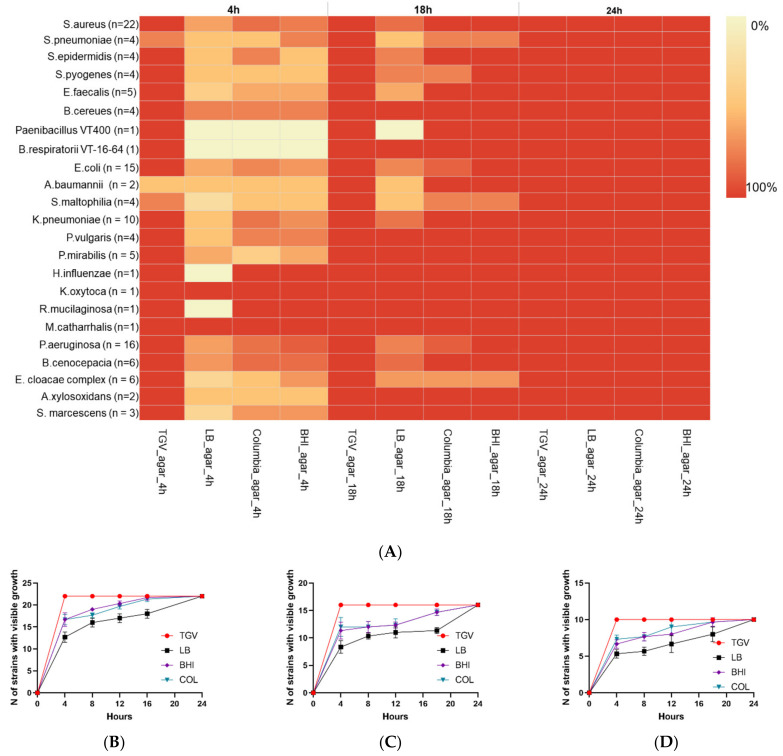
Comparative analysis of bacterial growth rate in different media (**A**) Growth rate is represented by a heat map, with each cell indicating the percentage of bacterial strains that showed growth at 4, 8, or 24 h. Red color intensity represents the highest rate of growth, whereas white color indicates no growth for a particular time period. (**B**) Number of *S. aureus* strains out of 22 tested that showed visible growth at different time periods on different media: TGV agar (TGV), LB agar (LB), Columbia agar (COL), brain heart infusion agar (BHI). (**C**) Number of *P. aeruginosa* strains out of 16 tested that showed visible growth at different time periods on TGV, LB, COL, or BHI. (**D**) Number of *K. pneumoniae* strains out of 10 tested that showed visible growth at different time periods on TGV, LB, COL, or BHI.

We first performed a validation of TGV agar by using monomicrobial cultures to confirm that it enables faster bacterial growth compared with that afforded by the conventional LB agar, Columbia agar, or brain heart infusion (BHI) agar. After 4 h of cultivation, visible growth was detected in 119 of 122 monomicrobial cultures (97.5%) in plates with TGV medium. Visible growth was not detected after 4 h only for *Stenotrophomonas maltophilia* (*n* = 1), *Streptococcus pneumoniae* (*n* = 1), and *Acinetobacter baumannii* (*n* = 1). However, these three strains were already visible after 8 h in culture (122/122; 100%) (Figure 1A).

Under the same conditions, visible growth on other agar media after 4 h of culturing was detected for fewer microorganisms. In particular, culturing on LB agar revealed growth of only 69 out of 122 strains (56.5%), whereas 89 out of 122 strains (72.9%) grew on Columbia agar and 87 out of 122 strains (71.3%) grew on BHI agar. Moreover, some *Streptococcus* spp. did not reveal clear growth even after 8 h on these media and became visible only by 24 h (Figure 1A). We also analyzed the effect of different media on the speed of growth of the key respiratory pathogens *Staphylococcus aureus*, *Pseudomonas aeruginosa*, and *Klebsiella pneumoniae* (Figure 1B–D). We found that TGV media enabled the fastest growth with 100% strains being visible within the first 4 h post plating, compared with lower numbers of positive bacterial growth detected in the plates filled with LB agar, Columbia agar, or BHI agar (*p* < 0.05).

**Figure 2:**
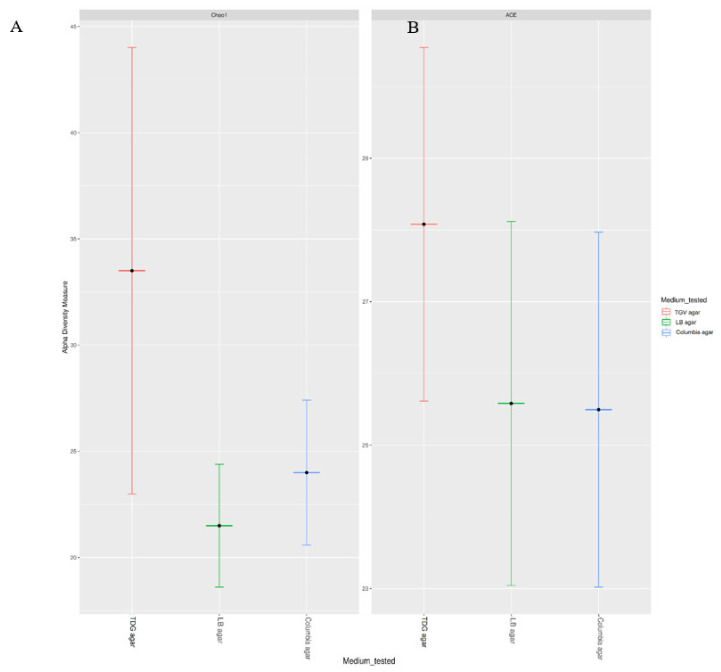
Increased diversity of bacterial species that showed growth on TGV medium, compared to that of species that grew on LB and Columbia agar, as revealed by the values of (**A**) Chao1 and (**B**) abundance-based coverage estimator (ACE) indices.

We observed higher species richness after growth for 4 h on TGV medium, as revealed by ACE and Chao 1 indices (28.07 and 33.5. respectively), compared with the values of these parameters after growth on LB agar (ACE = 25.6 and Chao 1 = 21.5) or Columbia agar (ACE = 25.5 and Chao 1 = 24; Figure 2A,B). Therefore, TGV was the only medium that allowed to detect visible growth of monomicrobial cultures after 4 h with high accuracy.

### 3.2. Estimation of the Diversity of Bacteria Grown from Patient Samples on TGV Medium

Next, we applied TGV medium for the study of polymicrobial infections in order to show that it enables the growth of a broader diversity of bacteria from clinical specimens. We used samples from patients with respiratory infections, which are usually associated with polymicrobial growth. Bacterial growth on TGV medium was seen within 4 h in 20 out of 20 clinical specimens (100%), whereas microbial growth was detected only in 8 out of 20 samples (40%) grown on LB agar that was used as standard medium. Only subsequent growth for 24 h revealed clear bacterial growth in all samples cultured in LB media.

Next, we analyzed polymicrobial growth in each sample by using TGV and LB media at 4 h and 24 h of culturing. A detailed description of bacteria that showed growth on different media is provided in:

After 4 h, direct plating on TGV media allowed the cultivatation of mixed bacterial communities in 19 of 20 clinical specimens (95%) (Table 1). The increase in culturing time to 24 h did not increase the diversity of the cultured bacteria. Under the same conditions, no mixed communities (0/100; 0%) were observed during exposure to LB media after 4 h. Subsequent culturing for up to 24 h allowed to identify polymicrobial communities only in 5 out of 20 samples (25%).

**Figure 3:**
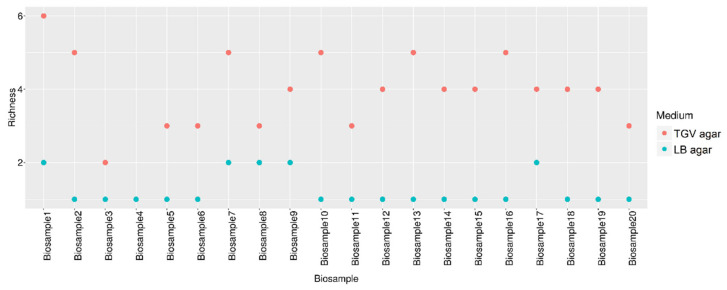
Comparison of the richness of bacteria that resulted in growth on TGV medium (after 4 h of culturing) compared with that of bacteria that showed growth (after 24 h) on LB agar (LB medium, *Burkholderia cepacia*-selective agar, chocolate agar, CHROMagar Staph aureus medium). Richness values for bio samples in two types of media are represented on a dot plot, displaying the distribution of numerical variables where each dot represents a value. The height of the column of dots represents the frequency for that value. The package ‘ggplot2’ within R (version 3.4.1) was used.

Next, we determined the overall gain of the identified microbial diversity that resulted in growth on TGV vs. standard media, demonstrating that even after 4 h of culturing, TGV uncovered a more diverse set of microorganisms from the biological specimens, compared with that revealed by the standard medium after 24 h of culturing (Figure 3).

Notably, in the majority of specimens grown on TGV, we identified more than one well-known pathogen of respiratory tract infections, whereas the standard method allowed the identification of one and, only sometimes, two pathogens. The highest microbial diversity was observed in sputum of the patients with cystic fibrosis (CF)—a disease that is known to be characterized by a particularly complex, mixed lung microbiome [68]. For example, from some tissue specimens, we isolated *P.aeruginosa, Staphylococcus haemolyticus, A. baumannii, Achromobacter xylosoxidans,* and *Burkholderia thailandensis* with TGV medium. Each of these microorganisms has been shown to be associated with lung infections in CF, whereas the standard method identified only the dominant *P. aeruginosa* [69,70,71].

The ability of TGV to enable growth of fastidious species is illustrated by the isolation of *Streptococcus milleri* from CF patient #10. This bacterium was only recently associated with pulmonary complications in CF because previously, *S. milleri* could not be cultured on standard laboratory media [72,73]. Another notable example was the identification with TGV of *K.pneumoniae*, *Rothia dentocariosa*, *Acinetobacter ursingii,* and *Bacillus pumilus*, in sample #18 from the patient with hospital-acquired pneumonia [74]. Each of these species had been shown previously to be associated with human infections. However, under the same conditions, the standard method identified only *K. pneumoniae*. A similar trend was noted for all other tested specimens from cases of other respiratory diseases whereby TGV enabled identification of a wider range of known respiratory pathogens compared to that revealed by the standard method.

Notably, by using TGV medium, we identified two previously unknown bacterial species *Chryseobacterium mucoviscidosis* sp. *nov*. and *Bacillus obstructivus* sp. nov. [75,76]. These bacteria have not been identified by MALDI-TOF, as they had very low similarity to known species, and their further whole genome sequencing proved that they were previously unknown bacterial species highly enriched in genes encoding pathogenic and antibiotic resistance factors.

### 3.3. Antibiotic Selection in Monomicrobial Cultures Using AtbFinder

A critical parameter for new methods intended to select antibiotics is accuracy with an acceptable number of false-positive or false-negative results, as compared to standard reference methods. Thus, we next evaluated the accuracy of AtbFinder’s performance for direct selection of 10 different antibiotics prescribed for the treatment of respiratory infections caused by Gram-positive or Gram-negative bacteria in a total of 122 monobacterial cultures after 4 h and 24 h of culturing. Cultures of 3 out of 122 strains (*S. pneumoniae* VT-SP-14, *S. maltophilia* VT-CFSM-4, and *A. baumannii* ATCC 17978) were excluded from the experiment due to the lack of growth in control wells after 4 h, leaving 1190 runs in total for analysis (119 bacterial species tested against 10 antibiotics; 1 run = each antibiotic tested against one bacterial strain). For the tested 119 strains, correct antibiotic selection, as gauged by the results of conventional AST, was achieved after 4 h in 1177 of 1190 runs (98.9%). In rare cases, false-positive and false-negative results were obtained. The overall sensitivity, specificity, PPV, and NPV were 99.6%, 98.1%, 98.5%, and 99.4%, respectively (Table 2 and Supplementary Table S2).

After 24 h of culturing, we preformed 1220 runs in total, as all bacteria at this time promoted growth on TGV control antibiotic-free medium. By 18 h, all microbial strains showed growth in control wells. Correct antibiotic selection was achieved at 1209 of 1220 runs (99.1%). The values of analyzed parameters after 24 h of culturing were marginally higher than those achieved after 4 h (sensitivity, 99.6%; specificity, 98.5%; PPV, 98.8%; and NPV, 99.4%; Table 2, Supplementary Table S3). Notably, there was no difference in the overall ability to detect Gram-positive and Gram-negative bacteria.

Next, we determined CA after 4 and 24 h of culturing for each antibiotic. After 4 h, the overall CA for all antibiotics used was 98.9% (1177/1190, 10 very major errors, 3 major errors). The rate of very major errors was 1.5% (10 very major errors/666 resistance outcomes), and the rate of major errors was 0.6% (3 major errors/512 susceptibility outcomes). The highest CA values were observed for levofloxacin, vancomycin (absolute agreement, 100%), followed by those for aztreonam, gentamicin, linezolid, piperacillin/tazobactam (for all: 118/119; 99.2%), amikacin, azithromycin, cefepime (for all: 117/119; 98.3%), and meropenem (116/119; 97.4%) (Table 3).

CA values for the AtbFinder were even higher when the period of culturing was extended. The overall CA for all antibiotics after 24 h of cultivation was 99.1% (1209/1220) with 8 very major errors and 3 major errors. The rate of very major errors was 1.2% (8 very major errors/688 resistance results), and the rate of major errors was 0.6% (3 major errors/521 susceptibility results). CA values became nominally higher for piperacillin/tazobactam and azithromycin. Thus, after 24 h of culturing, the absolute CA was observed for piperacillin/tazobactam, levofloxacin, and vancomycin with no errors detected (100%). Slightly lower, but nonetheless very high CA values were noted for aztreonam, azithromycin, gentamicin, linezolid (for all: 121/122; 99.2%), amikacin, cefepime (for all: 120/122; 98.3%), and meropenem (119/122; 97.5%) (Table 3).

At both time points tested, the level of major errors was very low, indicating that antibiotic-sensitive isolates did not grow on TGV medium with antibiotics added at chosen concentrations. In general, there was no discernible pattern of the errors that could be explained by the nature of microorganisms or antibiotics tested: AST errors occurred in various species and with various drugs. CA values were not statistically different between the antibiotics used.

### 3.4. Antibiotic Selection in Polymicrobial Cultures Using AtbFinder

When developing AtbFinder, we assumed that the antibiotic response of mixed cultures from the site of infection grown on TGV may be different compared to the antibiotic response of bacteria identified with the standard AST methods. We reasoned that if the antibiotic resistance profile of the principal pathogen identified with a standard AST does not properly reflect the collective antibiotic resistance, this circumstance can lead to a false-negative antibiotic selection.

**Figure 4:**
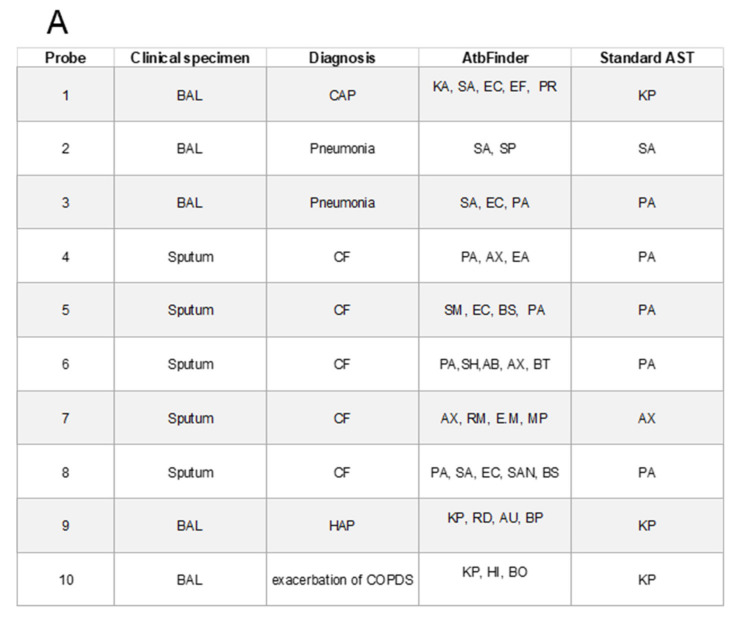

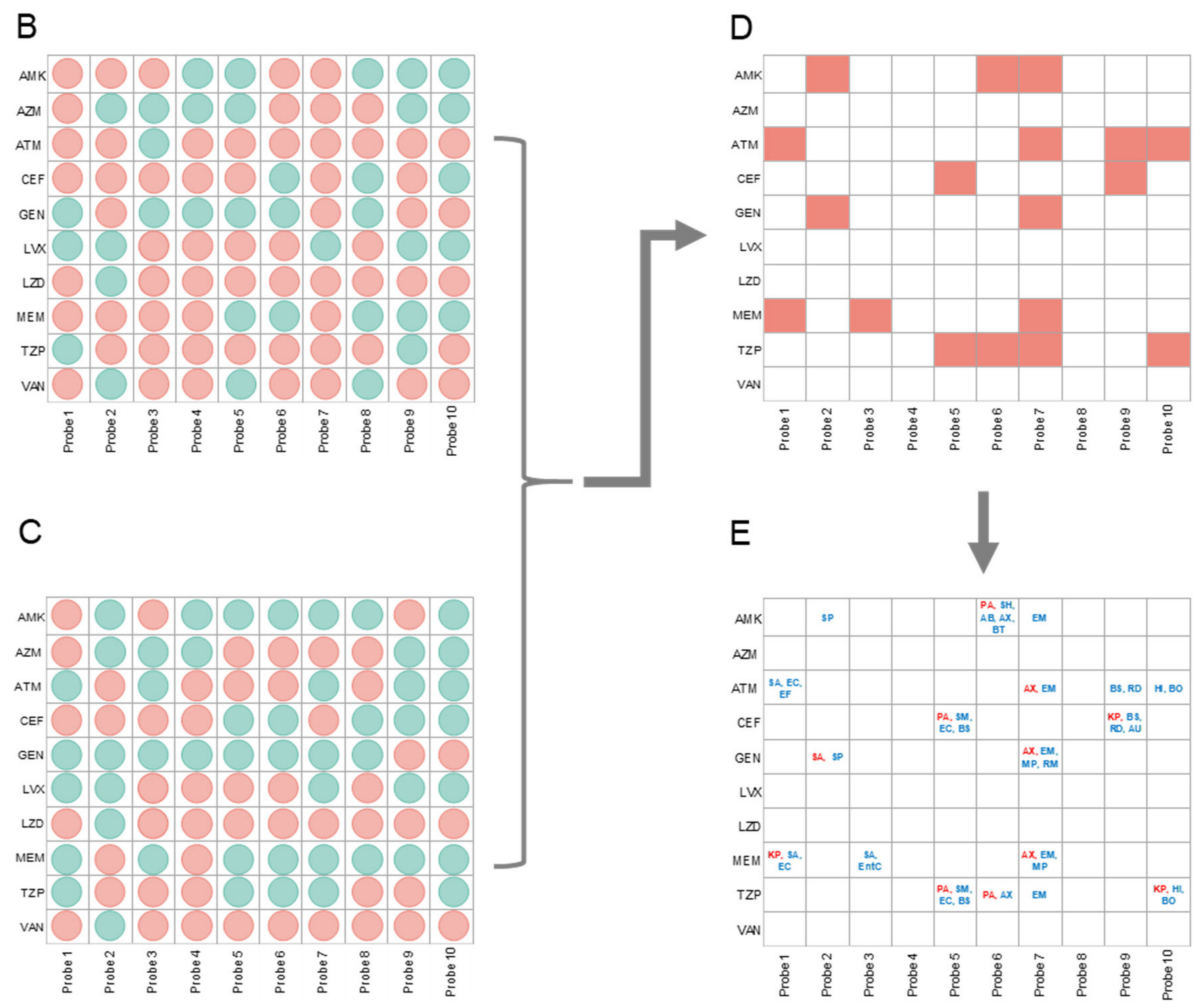
Selection of antibiotics for polymicrobial biological samples with AtbFinder compared with that achieved with a standard AST method.Antibiotics used: amikacin (AMK), azithromycin (AZM), aztreonam (ATM), cefepime (CEF), gentamycin (GEN), levofloxacin (LVX), linezolid (LZD), meropenem (MEM), piperacillin-tazobactam (TZP) vancomycin (VAN). (**A**) Comparison of the diversity of bacteria grown on TGV of AtbFinder (for 4 h) and on the standard medium used by AST (for 24 h). *A. baumannii* (AB)*, A. xylosoxidans* (AX), *A. ursingii* (AU), *Bacillus* spp. (BS), *Bacillus obstructivus* (BO), *B. pumilus* (BP), *B. sonorensis* (BS), *B. thailadensis* (BT), *Enterobacter cloacae* (EntC), *Enterococcus faecalis* (EF), *Elizabethkingia miricola* (EM), *Escherichia coli* (EC), *Haemophilus influenzae* (HI), *K. pneumoniae* (KP), *Microbacterium paraoxydans* (MP), *Proteus* spp. (PR), *R. mucilaginosa* (RM), *R. dentocariosa* (RD), *S. anginosus* (SAN), *S. aureus* (SA), *S. maltophilia* (SM), *S. heamolyticus* (SH), *S. pneumoniae* (SP). BAL—Bronchoalveolar lavage, CF—cystic fibrosis, CAP mommunity community-acquired pneumonia, HAP—Hospital-acquired pneumonia, COPD—Chronic obstructive pulmonary disease (**B**) Antibiotic efficacy identified with AtbFinder. (**C**) Antibiotic efficacy identified by the standard AST method. Red circles in (**B**) and (**C**) represent resistant determinations (“ineffective” antibiotics) and green circles represent susceptible determinations (“effective” antibiotics). (**D**) Analysis of the discrepancies in the efficacy of antibiotics between the AtbFinder and standard AST methods. Red squares depict antibiotics that according to AtbFinder were predicted to be “ineffective” for specific biosamples but deemed “effective” by the standard AST method. (**E**) Bacterial cultures from the discrepant cases outlined in that were suggested as ineffective against to be resistant to certain antibiotics with AtbFinder, and thus experienced growth in the wells with in the presence of that certain antibiotic, but were deemed “effective” against identified pathogens by the standard AST method were subcultured from the wells of AtFinder and identified. Bacteria marked with red letters were suggested to be the principal pathogen by the standard AST method and, when grown under the conditions modulated according to the standard AST method, were killed by this specific antibiotic (see Figure 4C). Bacteria marked in blue were not suggested to be the primary pathogen by the standard AST method.

To test this assumption, we used 10 randomly selected samples from the cases of respiratory infections previously tested in this study and compared the list of antibiotics selected as effective with AtbFinder (after growth for 4 h) (Figure 4B) to the list of antibiotics generated by the culture-based gold standard AST (microdilution after growth for 24 h) (Figure 4C). First, we observed a broader diversity of bacteria from the specimens grown on TGV of AtbFinder compared to the microbial diversity on LB agar used in the standard method (Figure 4A). Thus, along with the bacteria suggested as the primary pathogen by the standard AST, TGV enabled the growth of other bacteria, including well-known pathogens from the same biological specimens. Next, as expected, we identified a discrepancy between the antibiotics suggested as effective by the AtbFinder and standard AST (Figure 4D).

When the 10 biosamples were each tested against 10 antibiotics (100 evaluations in total), we found 18 discrepancies in antibiotic efficacy between those selected as effective with AtbFinder and the microbroth dilution method. In these cases, antibiotics were deemed as effective by the standard AST, but were suggested to be ineffective by AtbFinder, as the bacteria from those samples could grow in the wells of AtbFinder with those antibiotics (red squares in Figure 4D). Then, to identify the bacteria that grew in the wells of AtbFinder with these antibiotics, we performed their follow-up subculturing. As illustrated in Figure 4E, we were able to isolate different bacteria, including those that according to standard AST were suggested as the principal pathogen from 11 out of the total 18 discrepancy wells.

This result meant that in 11 out of 100 total antibiotic efficacy evaluations (11%), when a mixed bacterial community grew on AtbFinder, the principal pathogens resisted the antibiotics selected as effective in monobacterial cultures of these pathogens in conditions of the standard AST. Therefore, 11% of discrepancy cases potentially represents the rate of false positive results of the standard AST method and could mislead the selection of antibiotics that might be ineffective in certain patients. Most likely, this happened because complicated polymicrobial interactions at the site of infection that resulted in the higher resistance of bacteria, including the principal pathogen, were not adequately simulated by the standard method that utilizes monobacterial culture growth. However, such interactions could be modeled well with the AtbFinder system.

Notably, in some cases, narrow-spectrum antibiotics added to AtbFinder, active only against Gram-positive or Gram-negative bacteria, inhibited the growth of mixed bacterial communities formed by both Gram-positive and Gram-negative bacteria. For example, aztreonam was effective against mixed communities formed not only by the Gram-negative *P. aeruginosa*, but also by the Gram-positive *S. aureus* (in probe 3) (Figure 4A). Furthermore, vancomycin, known to be effective solely against Gram-positive bacteria, was effective against mixed communities comprised of not only Gram-positive bacteria, but also *P. aeruginosa*, *E. coli*, and other Gram-negative microorganisms (in probes 5, 8), indicating complicated interspecies interactions in polymicrobial communities when one bacteria is required to support the growth of other microorganisms (Figure 4A). This is in agreement with some recently published data that have shown that the overall response to antibiotics of a mixed community is sometimes the opposite to that of individual bacteria [77].

## 4. Discussion

New diagnostic methods for the selection of antibiotics for tailored empirical therapy and for the change of antibiotic therapy that failed in immunocompromised patients are urgently needed [78]. The availability of rapid and accurate methods of antibiotic selection for non-bloodstream infections, such as lung, urinary tract, skin, and soft tissue infections, would have a great impact on the disease outcome, length of hospital stay, possible complications, and spread of antibiotic resistance [79].

**Figure 5:**
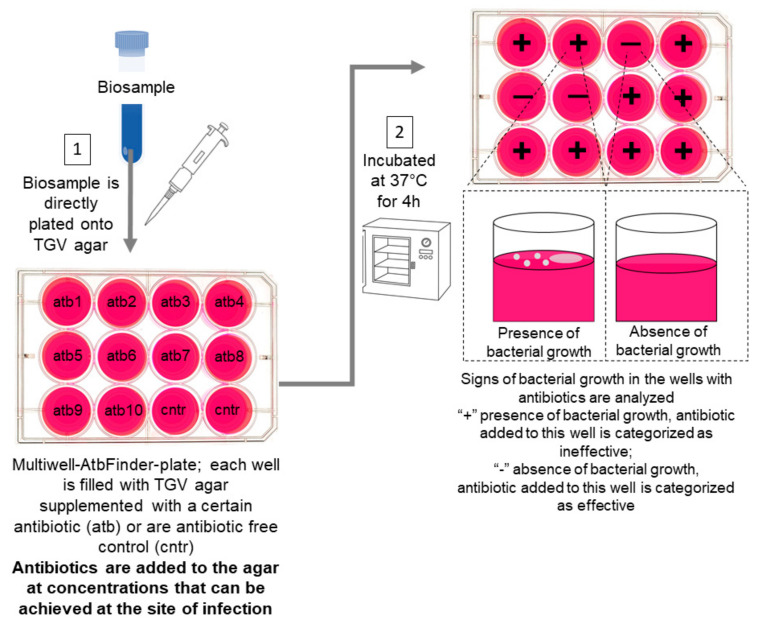
AtbFinder principle of operation and performance. Illustrated schematic of the AtbFinder performance protocol developed for different biosamples. (1) A biosample, which can be optionally diluted with sterile water, is plated onto the wells of 12–96-well AtbFinder plates. In the 12-well plates used in this study, ten “testing wells” contained TGV nutrient medium with antibiotics (one antibiotic per well) selected as per current therapeutic guidelines and added to the medium at concentrations clinically achievable at the site of infection. Two “control wells” contained antibiotic-free TGV medium. (2) Plate reading is performed following sampling and incubation at 37 °C for 4 h. The presence of microbial growth is identified with the naked eye and can be confirmed with a stereoscopic microscope. Microbial growth in any “testing well” means that in the pathological material, there are microorganisms resistant to the antibiotic that has been added to the nutrient medium in this well. This antibiotic is categorized as “ineffective”. The absence of bacterial growth in the well means that the antibiotic present in the well leads to a complete killing or inhibits the growth of all bacteria in the biospecimens and such an antibiotic is categorized as “effective”.

Our present experiments evaluated for the first time the performance of the novel AtbFinder system providing culture-based antibiotic selection within a short, 4 h period based on the novel principle to select antibiotics effective against polymicrobial communities from pathological material (Figure 5).

The AtbFinder method can be used for initial tailored empirical therapy and for the selection of an appropriate antibiotic to those patients who have not responded to the previous therapy and require change of antimicrobial treatment strategy. Our original hypothesis that the use of the AtbFinder system would provide a rapid and accurate selection of antibiotics for both monobacterial or polybacterial infections sampled directly from clinical specimens, was found to be confirmed experimentally. The AtbFinder system allowed faster growth of monobacterial cultures compared with that achieved by the standard method used in clinical diagnostics and demonstrated an increased richness with higher ACE and Chao 1 indices for bacteria that promoted growth within 4 h on AtbFinder medium than those recorded for bacteria grown on LB agar or Columbia agar (Figure 2).

Our study also revealed that the AtbFinder system provided accurate antibiotic selection compared to that achieved by the standard of care, with antibiotics added to the media at a concentration that can be achieved at the site of infection, with results being available after 4 h of culturing. If compared with the standard method that involves the isolation of pure bacterial cultures prior to AST, the AtbFinder system was faster by more than 32 h. The antibiotic selection was highly accurate already within 4 h (sensitivity, 99.6%; specificity, 98.1%; PPV, 98.5%; NPV, 99.4%, and CA, 98.9%); with no discernable bias in the error pattern toward Gram-positive or Gram-negative bacteria or toward a particular antibiotic class. Furthermore, the increase of culturing time from 4 h to 24 h did not significantly change the accuracy of the AtbFinder method performance, indicating that accurate results can be obtained after just several hours of culturing.

In the current study, TGV medium supported growth of a significantly more diverse set of bacteria from biological samples even after 4 h of culturing than the standard medium, broadly used in microbiological laboratories, after 24 h of culturing. Thus, the AtbFinder system allowed identification of polymicrobial cultures in 19 out of 20 samples after 4 h of culturing. This was in agreement with previous reports that emphasized the polymicrobial nature of the majority of respiratory tract infections [13]. Under the same conditions, the latter approach detected polymicrobial cultures in only 5 out of 20 samples. In this study, the standard method failed to detect not only some isolated commensal bacteria, but also well-known causative agents of respiratory infections. For example, AtbFinder allowed growth of *S. aureus, H. influenzae, E. miricola, A. baumannii, A. xylosoxidans*, and other well-known respiratory tract pathogens in samples from CF and chronic obstructive pulmonary disease. These microorganisms have been shown to cause recurrent respiratory infections but are completely overlooked by standard-of-care diagnostics.

In the current study, the AtbFinder system enabled growth of different complex mixtures of bacteria without a limit on the number of species that could be isolated. This finding is of primary importance due to two considerations. First, the presence of polymicrobial infections is one of the reasons for the inappropriate antibiotic selection and mortality of high-risk patients. The elimination of the dominant strains leads to the re-growth of other bacteria at the site of the infection unaffected by the initial antibiotic treatment, which may lead to disease progression and complications, e.g., due to the lack of sufficiently strong immune response. Moreover, as has been shown before, some bacteria that are considered nonpathogenic in the lungs of non-immunosuppressed subjects may be pathogenic in immunosuppressed hosts [80,81]. This is why, for some bloodstream infections, sterilization is widely used, particularly in patients with impaired immune response. Therefore, the fact that the AtbFinder system reliably detects both major and minor bacterial pathogens within the site of infection makes it promising for use in immunocompromised patients. Second, our assessment of antibiotic sensitivity within polymicrobial communities showed that in some cases, antibiotics selected with the standard method did not eliminate even the dominant bacteria in mixed microbial communities. Such false-negative data might result in the selection of ineffective antibiotics and therapy failure [82]. The reason for false-positive results generated by the gold standard method may be because this method enables growth of monobacterial cultures but does not take into consideration higher tolerance to antibiotics of bacteria within mixed communities [15]. However, “real life” infections are predominantly polymicrobial by nature [10]. The resulting survival of the lead pathogen leads to its re-growth and therapy failure. According to our in vitro data, AtbFinder is less prone to such limitations.

Furthermore, the use of the AtbFinder system for the treatment of polybacterial infections allowed selection of not only broad spectrum but also narrow spectrum antibiotics, confirming the presence of complexed interspecies interactions in mixed bacterial communities.

Notably, by using AtbFinder, we have isolated previously unknown bacterial species *Chryseobacterium mucoviscidosis* sp. nov. and *Bacillus obstructivus* sp. nov. that possessed several typical virulence factors, such as hemolysins and others, which are found in other respiratory pathogens [76,77,83]. Moreover, in these bacteria, we identified several antibiotic resistance genes that, if found in endospore-forming *Bacillus* spp., are of concern because of the possible spread of antibiotic resistance among sporobiota members [84]. The identification of previously unknown bacteria as well as some fastidious species, such as *S. milleri*, indicates that the developed TGV medium provides unique growth factors and cultivation environment that are not achievable with the standard media [19,85].

The use of the AtbFinder system enables to quickly select antibiotics that are effective for the treatment of each particular condition (i.e., those that kill or inhibit bacterial growth of all microorganisms present in the clinical specimen) without the need to identify precisely the causative agents of the infection or to determine MICs. Importantly, although the AtbFinder test does not allow immediate identification of bacteria on the species level, bacterial cultures that promoted growth on TGV medium can be isolated and subsequently identified by using culture-based techniques and any other standard method for the study of bacteria.

The AtbFinder system has all benefits of phenotypic culture-based methods, but is 30–54 h faster than standard culture-based diagnostics that require time-consuming isolation of pure bacterial cultures. Even compared with direct AST, the AtbFinder method allows antibiotic selection on a much shorter timescale [60]. Like direct AST, the AtbFinder system enables direct sampling of biological specimens without the need for culturing or time-consuming sample processing. Furthermore, the AtbFinder method can indicate suitable antibiotics in only 4 h, whereas direct AST requires 18–36 h (and that is why direct AST is not applicable for tailoring empirical antibiotic therapy) [86,87,88].

Despite being as fast as some molecular biology methods, the AtbFinder system lacks main disadvantages of molecular methods based on next-generation sequencing and *16S RNA* sequencing, such as overestimation of antimicrobial resistance and inability to inform on antimicrobial susceptibility [37,38]. Moreover, there is no need for either specific equipment (except a thermostat), or highly trained personnel, because the presence or absence of bacterial growth can be identified by personnel with basic laboratory skills. In this study, we used the AtbFinder system with a set of antibiotics for the treatment of respiratory infections. However, it can easily be adjusted for the diagnosis of infections of other parts of the body, such as urinary tract, skin, or soft tissues, by including the antibiotics used for the therapy of these infections in TGV medium. Moreover, it can be used for the cultivation of bacteria with different oxygen requirements. The presence of bacterial growth was analyzed with the naked eye and stereoscopic microscopy, but a more sophisticated device for visual monitoring can be used to increase analysis accuracy.

One of the limitations of the current study was that we used antibiotics added to the media at a concentration that can be achieved at the site of infection based on the literature data. Although this concentration is true for the majority of patients, such an approach might not represent the entire patient population, particularly those with underlying kidney or liver diseases known to be associated with notable pharmacokinetic alterations [89,90].

We believe that the AtbFinder system may become a novel and valuable tool in improving antibiotic selection, with as little as 4 h turnaround time. In terms of possible laboratory implementation, AtbFinder dramatically shortens the testing routine by allowing appropriate antibiotic selection on the same day. Therefore, AtbFinder enables more effective antibiotic selection for targeted empirical therapy and accurate antibiotic replacement, especially in high-risk immunocompromised patients. Future studies will be necessary to investigate the clinical efficacy, sensitivity, and accuracy of the AtbFinder system; and the clinical impact of its use alone and as an auxiliary method for standard diagnostics.

## Figures and Tables

**Table 1:** Comparison of diversity values of bacteria grown on AtbFinder and standard media for 4 and 24 h.

#	Clinical Specimen	Diagnosis	Bacteria that Showed Growth on TGV Medium		Bacteria Identified with Standard Media *	
			4 h	24 h	4 h	24 h
1	BAL	COPD	*Burkholderia gladioli* *Neisseria* spp. *Haemophilus parainfluenzae* *S. mitis* *R. mucilaginosa* *Streptomyces violaceruber*	*B. gladioli* *Neisseria* spp. *H. parainfluenzae* *S. mitis* *R. mucilaginosa* *S. violaceruber*	*-*	*S. mitis* *R. mucilaginosa*
2	BAL	COPD	*S. aureus* *K. pneumoniae* *E. coli* *E. faecalis* *Proteus* spp.	*S. aureus* *K. pneumoniae* *E. coli* *E. faecalis* *Proteus* spp.	*K. pneumoniae*	*K. pneumoniae*
3	BAL	CAP	*S. aureus* *S. pneumoniae*	*S. aureus* *S. pneumoniae*	*-*	*S. aureus*
4	BAL	CAP	*S. aureus*	*S. aureus*	*S. aureus*	*S. aureus*
5	BAL	CAP	*P. aeruginosa* *H. influenza* *M. catarrhalis*	*P. aeruginosa* *H. influenza* *M. catarrhalis*	*P. aeruginosa*	*P. aeruginosa*
6	BAL	VAP	*S. aureus* *E. cloacae* *P. aeruginosa*	*S. aureus* *E. cloacae* *P. aeruginosa*	*-*	*P. aeruginosa*
7	BAL	VAP	*S. aureus* *K. pneumoniae* *E. coli* *C. mucoviscidosis* *S. aureus*	*S. aureus* *K. pneumoniae* *E. coli* *C. mucoviscidosis* *S. aureus*	*-*	*S. aureus* *E. coli*
8	Sputum	CF	*P. aeruginosa* *S. aureus* *R. mucilaginosa*	*P. aeruginosa* *S. aureus* *R. mucilaginosa*	*P. aeruginosa*	*P. aeruginosa* *S. aureus*
9	Sputum	CF	*P. aeruginosa* *S. aureus* *S. maltophilia* *B. cepacia*	*P. aeruginosa* *S. aureus* *S. maltophilia* *B. cepacia*	*-*	*P. aeruginosa* *B. cepacia*
10	Sputum	CF	*P. aeruginosa* *Micrococcus luteus* *S. aureus* *S. oralis* *S. milleri*	*P. aeruginosa* *M. luteus* *S. aureus* *S. oralis* *S. milleri*	*-*	*P. aeruginosa*
11	Sputum	CF	*P. aeruginosa* *A. xylosoxidans* *E. coli*	*P. aeruginosa* *A. xylosoxidans* *E. coli*	*P. aeruginosa*	*P. aeruginosa*
12	Sputum	CF	*S. maltophilia* *E. coli* *Bacillaceae* spp. *P. aeruginosa*	*S. maltophilia* *E. coli* *Bacillaceae* spp. *P. aeruginosa*	*P. aeruginosa*	*P. aeruginosa*
13	Sputum	CF	*P. aeruginosa* *S. haemoliticus* *A. baumannii* *A. xylosoxidans* *B. thailandensis*	*P. aeruginosa* *S. haemoliticus* *A. baumannii* *A. xylosoxidans* *B. thailandensis*	*-*	*P. aeruginosa*
14	Sputum	CF	*B. multivorans* *Corynebacterium pseudodiphtheriticum* *Moraxella catharrhalis* *Paenibacillus pabuli*	*B. multivorans* *C. pseudodiphtheriticum* *M. catharrhalis* *P. pabuli*	* - *	*C. pseudodiphtheriticum*
15	Sputum	CF	*A. xylosoxidans* *R. mucilaginosa* *Elizabethkingia miricola* *Microbacterium paraoxydans*	*A. xylosoxidans* *R. mucilaginosa* *E. miricola* *M.* *paraoxydans*	*-*	*A. xylosoxidans*
16	Sputum	CF	*P. aeruginosa* *S. aureus* *E. coli* *S. anginosus* *B. sonorensis*	*P. aeruginosa* *S. aureus* *E. coli* *S. anginosus* *B. sonorensis*	*P. aeruginosa*	*P. aeruginosa*
17	Sputum	CF	*P. aeruginosa* *S. aureus* *E. coli* *B. cereus*	*P. aeruginosa* *S. aureus* *E. coli* *B. cereus*	*P. aeruginosa*	*P. aeruginosa* *S. aureus*
18	BAL	HAP	*K. pneumoniae* *Rothia dentocariosa* *A.ursingii* *Bacillus pumilus*	*K. pneumoniae* *R. dentocariosa* *A. ursingii* *B. pumilus*	*-*	*K. pneumoniae*
19	BAL	CAP	*H. influenzae* *Actinobacillus suis* *Eikenella corrodens* *Actinomyces oris*	*H. influenzae* *A. suis* *E. corrodens* *A.oris*	*-*	*H. influenzae*
20	BAL	COPD	*K. pneumoniae* *H. influenza* *Bacillus obstructivus*	*K. pneumoniae* *H. influenza* *Bacillus obstructivus*	*-*	*K. pneumoniaee*

* Bacteria from cystic fibrosis patient samples were plated on LB medium as well as cultivated on *Burkholderia cepacia* selective agar, chocolate agar, and CHROMagar Staph aureus medium.

**Table 2:** Overall identification performance of the AtbFinder system after 4 and 24 h of culturing.

Species	N True Positive		N True Negative		N False Positive		N False Negative		Sensitivity (%)		Specificity (%)		PPV (%)		NPV (%)	
	4 h	24 h	4 h	24 h	4 h	24 h	4 h	24 h	4 h	24 h	4 h	24 h	4 h	24 h	4 h	24 h
*S. aureus*	134	134	84	84	1	1	1	1	99.3	99.3	98.8	98.8	99.3	99.3	98.8	98.8
*S. pneumoniae*	12	16	18	24	0	0	0	0	100	100	100	100	100	100	100	100
*S. epidermidis*	16	17	23	23	1	0	0	0	100	100	95.8	100	94.1	94.1	100	100
*S. pyogenes*	10	10	30	30	0	0	0	0	100	100	100	100	100	100	100	100
*E. faecalis*	27	27	22	22	1	1	0	0	100	100	95.6	95.6	96.4	96.4	100	100
*B. cereues*	17	17	23	23	0	0	0	0	100	100	100	100	100	100	100	100
*Paenibacillus* VT400	5	5	4	4	1	1	0	0	100	100	80	80	83.3	83.3	100	100
* B. respiratorii * VT1664	3	3	7	7	0	0	0	0	100	100	100	100	100	100	100	100
*E. coli*	70	71	78	78	2	1	0	0	100	100	97.5	98.8	97.2	97.2	100	100
*A. baumannii*	8	16	1	4	0	0	0	0	100	100	100	100	100	100	100	100
* S. maltophilia *	19	28	10	11	1	1	0	0	100	100	90.1	91.6	95	95	100	100
*K. pneumoniae*	51	51	47	47	1	1	1	1	98.1	98.1	97.9	97.9	98.1	98.1	97.9	97.9
*P. vulgaris*	18	18	22	22	0	0	0	0	100	100	100	100	100	100	100	100
*P. mirabilis*	22	22	28	28	0	0	0	0	100	100	100	100	100	100	100	100
*H. influenzae*	2	2	8	8	0	0	0	0	100	100	100	100	100	100	100	100
*K. oxytoca*	6	6	4	4	0	0	0	0	100	100	100	100	100	100	100	100
*R. mucilaginosa*	4	4	6	6	0	0	0	0	100	100	100	100	100	100	100	100
*M. catharrhalis*	4	4	6	6	0	0	0	0	100	100	100	100	100	100	100	100
*P. aeruginosa*	122	122	35	35	2	2	1	1	99.2	99.2	94.6	94.6	98.4	98.4	97.2	97.2
*B. cenocepacia*	53	53	7	7	0	0	0	0	100	100	100	100	100	100	100	100
*E. cloacae complex*	31	31	29	29	0	0	0	0	100	100	100	100	100	100	100	100
*A. xylosoxidans*	16	16	4	4	0	0	0	0	100	100	100	100	100	100	100	100
*S. marcescens*	14	14	16	16	0	0	0	0	100	100	100	100	100	100	100	100
Gram-positive bacteria	403	408	259	265	6	5	2	2	99.9	99.9	96.8	97.2	97.4	97.4	99.6	99.6
Gram-negative bacteria	437	455	295	298	14	14	2	2	99.8	99.8	98.6	98.8	99.2	99.2	99.7	99.7

NPV, negative predictive value; PPV, positive predictive value.

**Table 3:** Category agreement of antibiotic sensitivity results of the AtbFinder method with those obtained by the standard method after 4 and 24 h of culturing.

Antibiotic	Category Agreement: No of Correct Identifications/Total No of Observations (%)		No of Errors			
			Very Major (False-Positive)		Major (False-Negative)	
	4 h	24 h	4 h	24 h	4 h	24 h
Piperacillin-tazobactam	118/119 (99.2)	122/122 (100)	1	0	0	0
Meropenem	116/119 (97.4)	119/122 (97.5)	2	2	1	1
Levofloxacin	119/119 (100)	122/122 (100)	0	0	0	0
Aztreonam	118/119 (99.2)	121/122 (99.2)	1	1	0	0
Gentamicin	118/119 (99.2)	121/122 (99.2)	0	0	1	1
Amikacin	117/119 (98.3)	120/122 (98.3)	2	2	0	0
Azithromycin	117/119 (98.3)	121/122 (99.2)	2	1	0	0
Vancomycin	119/119 (100)	122/122 (100)	0	0	0	0
Cefepime	117/119 (98.3)	120/122 (98.3)	2	2	0	0
Linezolid	118/119 (99.2)	121/122 (99.2)	0	0	1	1
Overall performance	1177/1190 (98.9)	1209/1220 (99.1)				

Bacterial cultures included monomicrobial cultures of *Staphylococcus aureus* (*n* = 22), *Streptococcus pneumoniae* (*n* = 4), *Streptococcus epidermidis* (*n* = 4), *Streptococcus pyogenes* (*n* = 2), *Enterococcus faecalis* (*n* = 5), *Bacillus cereues* (*n* = 4), *Paenibacillus* VT400 (*n* = 1), Bacillus respiratorii VT-16–64 (*n* = 1) *Escherichia coli* (*n* = 15), *Acinetobacter baumannii* (*n* = 2), *Stenotrophomonas maltophilia* (*n* = 4), *Klebsiella pneumoniae* (*n* = 10), *Proteus vulgaris* (*n* = 4), *Proteus mirabilis* (*n* = 5), *Haemophilus influenzae* (*n* = 1), *Klebsiella oxytoca* (*n* = 1), *Rothia mucilaginosa* (*n* = 1), *Moraxella catharrhalis* (*n* = 1) *Pseudomonas aeruginosa* (*n* = 16), *Burkholderia cenocepacia* (*n* = 6), *Enterobacter cloacae complex* (*n* = 6), *Achromobacter xylosoxidans* (*n* = 2), *Serratia marcescens* (*n* = 3).

## Supplementary Materials

The following are available online at https://www.mdpi.com/article/10.3390/microorganisms9050990/s1, Supplementary Table S1. Individual patterns of resistance to antibiotics. Supplementary Table S2. Results of antimicrobial susceptibility testing by using the AtbFinder system (4 h culturing) and standard method (24 h culturing) for 119 Gram-positive and Gram-negative pathogens. Supplementary Table S3. Results of antimicrobial susceptibility testing by using the AtbFinder system and standard method for 122 Gram-positive and Gram-negative pathogens after 24 h of culturing.
